# Effect of Different Expression of Immune-Related lncRNA on Colon Adenocarcinoma and Its Relation to Prognosis

**DOI:** 10.1155/2020/6942740

**Published:** 2020-06-06

**Authors:** Meiwei Mu, Yi Tang, Zheng Yang, Yuling Qiu, Xiaohong Li, Wuning Mo, Qisheng Su

**Affiliations:** ^1^Department of Clinical Laboratory, First Affiliated Hospital of Guangxi Medical University, Nanning, Guangxi Zhuang Autonomous Region, China; ^2^College of Basic Medicine, Guangxi Medical University, Nanning, Guangxi Zhuang Autonomous Region, China

## Abstract

**Objective:**

To explore the expression of immune-related lncRNAs in colon adenocarcinoma and find out the effect on how these lncRNAs influence the development and prognosis of colon adenocarcinoma.

**Method:**

Transcriptome data of colon adenocarcinoma from The Cancer Genome Atlas (TCGA) were downloaded, and gene sets “IMMUNE RESPONSE” and “IMMUNE SYSTEM PROCESS” were sought from the Molecular Signatures Database (MSigDB). The expression of immune-related genes was extracted that were immune-related mRNAs. Then, the immune-related lncRNAs were sought out by utilizing of the above data. Clinical traits were combined with immune-related lncRNAs, so that prognostic-related lncRNAs were identified by Cox regression. Multivariate Cox regression was built to calculate risk scores. Relationships between clinical traits and immune-related lncRNAs were also calculated.

**Result:**

A total of 480 colorectal adenocarcinoma patients and 41 normal control patients' transcriptome sequencing data of tissue samples were obtained from TCGA database. 918 immune-related lncRNAs were screened. Cox regression showed that 34 immune-related lncRNAs were associated with colon adenocarcinoma prognosis. Seven lncRNAs were independent risk factors.

**Conclusion:**

This study revealed that some lncRNAs can affect the development and prognosis of colon adenocarcinoma. It may provide new theory evidence of molecular mechanism for the future research and molecular targeted therapy of colon adenocarcinoma.

## 1. Background

Colon adenocarcinoma (COAD) is the third most common cancer in the gastrointestinal tract. Most COAD are sporadic and have no genetic or family history. Though the research of the molecular mechanism and pathway has been making some progress, there is no perfect explanation on the origin, development, and metastasis of COAD [1–3]. Long noncoding RNA (lncRNA) is a class of RNAs with a length of more than 200 bases, lacking complete open reading frames. Studies have shown that lncRNA can regulate gene expression at multiple levels, such as transcription, translation, regulation of protein modification, and RNA proteins [4]. lncRNAs influence cellar physiology and pathology, and they are involved in the tumor development [5]. Some former researches found a number of lncRNAs involved in the development of many diseases: SCHLAP1 promotes proliferation and metastasis of prostate cancer by targeting miR-198 and promoting the MAPK1 pathway [6]. And some diseases, such as melanoma, have satisfactory results which are presented in the treatment [7]. In COAD, CASC15 demonstrated to have promoted growth and metastasis through the activation of the Wnt/*β*-catenin signaling pathway in a miR-4310/LGR5-dependent manner [8]. And ROR1-AS1 level in COAD tissues was remarkably higher than that in normal tissues [9].

In this study, we attempted to explore the effect of lncRNAs, regulating immune-related processes, on the survival of colorectal adenocarcinoma patients. Based on these lncRNAs, we constructed a model to predict the risk of death of COAD patients. We found out that the relationship between these immune-related lncRNAs and prognosis of COAD are of great importance and may be helpful in building a new way to treat COAD.

## 2. Materials and Methods

### 2.1. Acquisition of Colon Adenocarcinoma Data from TCGA

COAD transcriptome expression data in the format of Fragments Per Kilobase Million (FPKM) of The Cancer Genome Atlas (TCGA) were downloaded from Genomic Data Commons (GDC, https://gdc.cancer.gov/), which are open to the public. Clinical traits were also downloaded. Among these, there were 480 colorectal adenocarcinoma patients and 41 normal control patients.

### 2.2. Acquisition of Immune-Related Genes from MSigDB

In order to extract immune-related lncRNAs, two gene sets “IMMUNE RESPONSE” and “IMMUNE SYSTEM PROCESS” were downloaded from the Molecular Signatures Database (MSigDB, https://www.gsea-msigdb.org/gsea/msigdb/). Then, immune-related mRNA expression was extracted from the above two gene sets.

### 2.3. Immune-Associated lncRNAs

Through Pearson's method, correlations between the mRNAs and lncRNAs were calculated. lncRNAs with the absolute value of correlation coefficient > 0.4 and *p* < 0.001 were considered as immune-related lncRNAs for further analysis.

### 2.4. Cox Regression

Immune-related lncRNA expression matrix was merged with clinical traits. Univariate Cox regression was performed on the above data to extract immune-related lncRNAs which are associated with survival time, and *p* < 0.01 was used as threshold. All these lncRNAs were divided into two categories: high-risk lncRNAs with HR > 1 and low-risk lncRNAs with HR < 1. A stepwise multivariate Cox regression was conducted. The risk score of each patient was calculated according to the multivariate Cox regression. The formula can be expressed as follows: risk score = *β*gene1 × Expressiongene1 + *β*gene2 × Expressiongene2 + *β*gene3 × Expressiongene3 + ⋯+*β*genen × Expressiongenen. Patients were divided into two groups according to the median value of risk scores. If the risk score was higher than the median, it was assigned to the high-risk group; otherwise, it was assigned to the low-risk group. The survival curve and risk curve were drawn based on risk scores and survival information.

### 2.5. Independent Prognostic Analysis

Comparing clinical traits and risk scores with survival status and survival time, the risk score was determined whether it can be used as an independent prognostic factor. Single factor and multifactorial analysis were performed to judge if these factors can be used as independent prognostic factors. The receiver operator characteristic (ROC) curve was drawn to determine whether the result was reliable.

### 2.6. Clinical Relevance Analysis

A box chart was drawn to analyze whether clinical traits were associated with lncRNAs in the risk prediction model.

## 3. Result

### 3.1. Prognosis-Related Immune lncRNA

**Figure 1:**
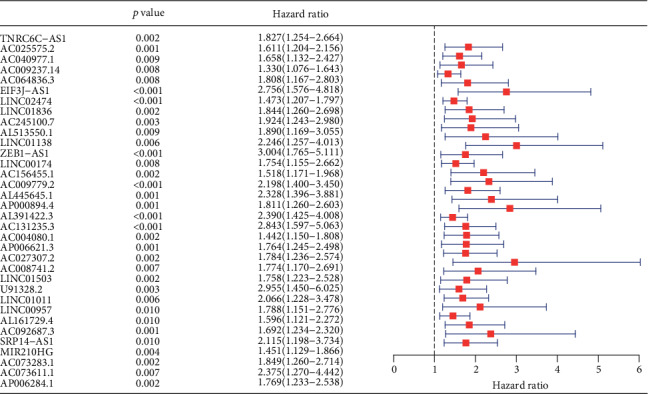
Univariate Cox regression showed that 34 immune-related lncRNAs were risk factors for COAD patients (hazard ratio > 1).

329 immune-related mRNAs were obtained. 918 immune-related lncRNAs were obtained through Pearson's correlation. The expression of immune-related lncRNAs were extracted and fused with clinical survival data. 34 immune-related lncRNAs were associated with the survival of COAD (*p* < 0.01). Among them, all of these prognosis-related lncRNAs were classified as high risk factors (Figure 1).

### 3.2. Cox Regression

**Figure 2:**
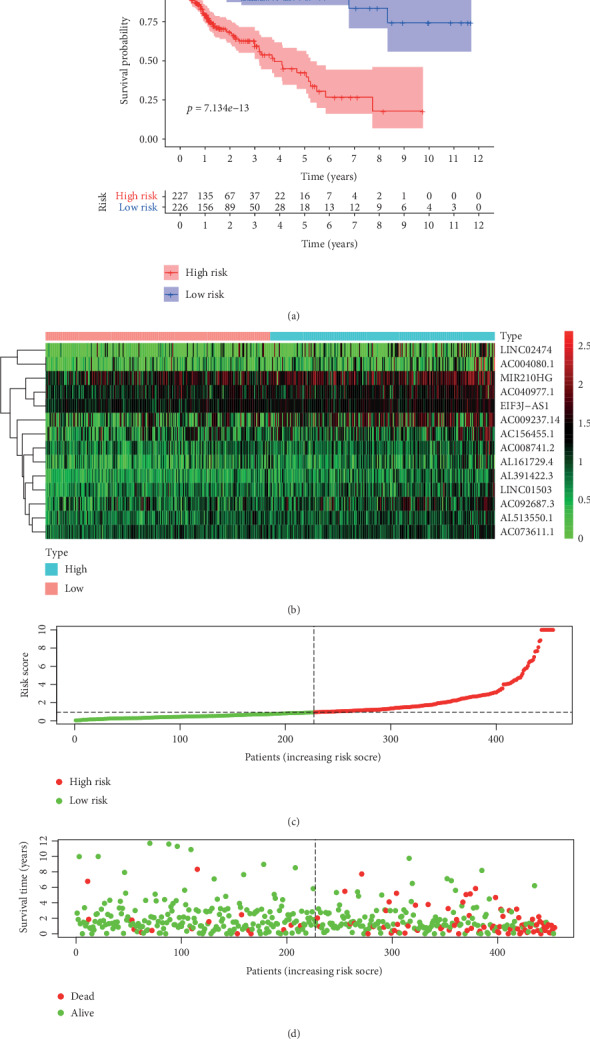
The survival curve shows that patients in the high-risk score group have a shorter survival time than those in the low-risk score group (a). The heat map of prognosis-related lncRNAs in COAD (b). Risk curves were drawn based on the data of 480 patients. Take the risk scores as the vertical coordinate and the patient data as the horizontal coordinate. We can see the risk scores were increasing, in which the low-risk curve was in green whereas the high-risk curve was in red (c). Green dots mean people who were still alive, and red dots mean people who were already dead. As the risk scores were increasing, the deaths were added; the red dots were concentrated in the high-risk area. And in the low-risk area, where the green dots were concentrated, patients are almost still alive (d).

Based on the above prognosis-related immune lncRNAs, the Cox regression model was built (Table 1). Next, all risk scores were calculated according to the Cox regression model. All COAD patients were divided by the median value of risk scores: high-risk group and low-risk group. According to the two groups, survival curves were drawn. The chart showed that the survival probability of the low-risk group was higher than that of the high-risk group. The chart below confirmed this conclusion, because patients' survival time in the high-risk group was evidently lower than that in the low-risk group. The number of deaths in the high-risk group increased faster than that in the low-risk group. By 11th year, only the patients in the low-risk group still survived (Figure 2).

### 3.3. Independent Prognostic Analysis

**Figure 3:**
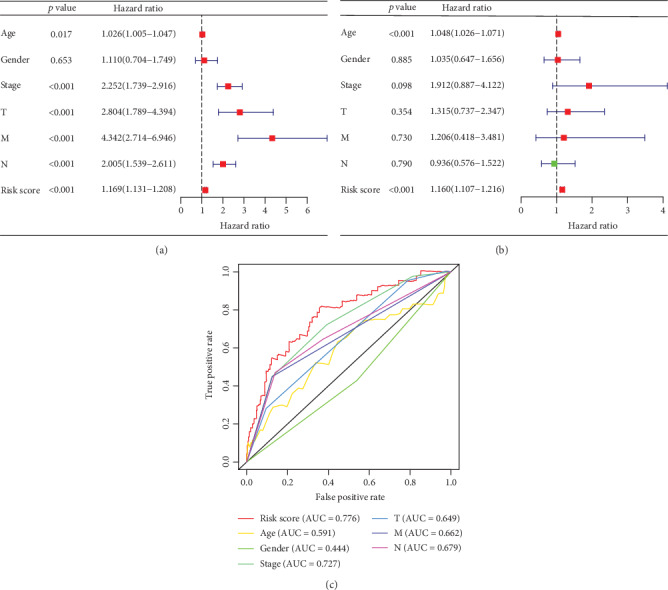
Univariate Cox regression showed that age, clinical stage, T stage, N stage, M stage, and risk score were all correlated with the prognosis of COAD patients (a). Multivariate Cox regression showed that only the risk score was an independent risk factor for COAD (b), and the ROC curve showed that the risk score had the largest area under the curve (c).

In univariate Cox regression, clinical stage, T stage, M stage, N stage, and risk score were thought to be associated with survival time in COAD patients (*p* < 0.001). Among them, age and risk score were independent prognostic factors in multivariate Cox regression (*p* < 0.001), but gender, clinical stage, T stage, M stage, and N stage were not. The ROC was drawn, and the area under the curve (AUC) was calculated to confirm the reliability of this conclusion. The area under the curve of gender was 0.444; all of the other factors including the risk score, age, clinical stage, T stage, M stage, and N stage were over 0.500. Among them, the AUC of risk score was 0.776 (Figure 3).

### 3.4. Clinical Relevance Analysis

**Figure 4:**
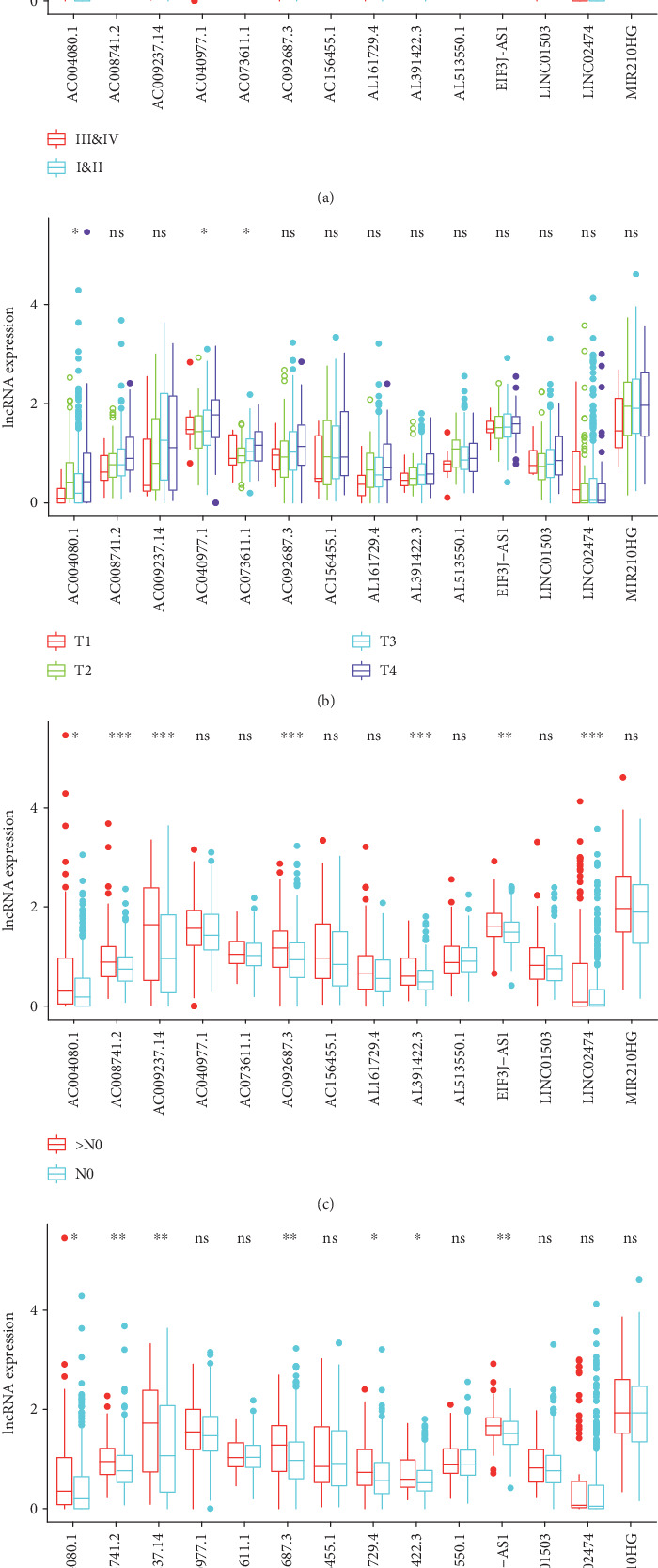
Multiple immune-related lncRNAs were associated with clinical traits (∗ means they had relevance, and the more they had, the greater the correlation; “ns” means they had no relevance). (a) Relationship between immune-related lncRNAs and clinical stages. (b) Relationship between immune-related lncRNAs and T stages. (c) Relationship between immune-related lncRNAs and N stages. (d) Relationship between immune-related lncRNAs and M stages.

A box chart was drawn to analyze whether lncRNAs are associated with clinical traits. The following results were obtained: T stage was associated with AC004080.1, AC040977.1, and AC073611.1; M stage was associated with AC004080.1, AC008741.2, AC009237.14, AC092687.3, AL161729.4, 29.4AL391422.3, and EIF3J−AS1 3; N stage was associated with AC004080.1, AC008741.2, AC009237.14, AC092687.3 AL391422.3, EIF3J−AS1, and LINC02474 4; and clinical stage was associated with AC008741.2, AC009237.14, AC092687.3, AC156455.1, AL391422, EIF3J−AS, and LINC0247 (Figure 4).

## 4. Discussion

The usual treatment of COAD is surgical treatment, which still has the insufficiency. In recent years, emerging immunotherapy methods such as PD-1/PD-L1 inhibitor which targeted at immune checkpoints have developed rapidly in the treatment of tumor and achieved a certain curative effect [10, 11]. These indicate that immune intervention has a broad application prospect in tumor therapy. However, there is much room for improvement in the management of colon cancer at present, and the prognosis of patients with advanced COAD is still very limited by the existing treatments. Therefore, new strategies for the treatment and management of COAD are necessary. Based on these current situations, we attempted to analyze the transcriptome sequencing data of COAD in TCGA, a public database. Because lncRNAs play a wide range of regulatory roles in various biological processes of the organism, we focused our research on lncRNAs and tried to correlate with immunity. We speculate that immune-related lncRNAs may be involved in important regulation in the immune process of the body against COAD.

In this study, we extracted the expression amount of lncRNAs regulating immune gene expression in COAD patients. The results of univariate Cox analysis showed that 34 immune-related lncRNAs were significantly associated with the survival time of patients with COAD, which is consistent with our view that lncRNAs may be involved in the regulation of the body's antitumor immune process. The results of multivariate Cox regression showed that the formula containing 14 immune-related lncRNA expressions could well distinguish patients with good prognosis from those with poor prognosis. This index has advantages over widely used clinical stages, T stage, N stage, M stage, and age. We believe that this formula can improve the management of patients with COAD.

Among 14 immune-related lncRNAs, EIF3J-AS1 was reported to promote COAD cell proliferation and inhibit apoptosis [12]. Our study found that EIF3J-AS1 was associated with the expression of immune genes in COAD, and its high expression predicted shorter survival time. LINC01503 and LINC02474 were confirmed to be associated with the survival of COAD in bioinformatics-based studies [13, 14], and LINC01503 could regulate the expression of miR4492/FOXK1 signaling which promotes proliferation and invasion of cells of COAD [15]. MIR210HG has been experimentally demonstrated to promote proliferation and invasion of tumor cells in hepatocellular carcinoma, non-small-cell lung cancer, breast cancer, cervical cancer, osteosarcoma, and other tumors. Its high expression is associated with poor prognosis of cancer patients [16–20]. He et al. and Ruan et al. found that they also predicted poor prognosis in COAD [21, 22], which is consistent with the results of our study, suggesting that our prediction of their survival in COAD is reliable. The remaining lncRNAs have not been studied in COAD to our knowledge, and we found that they are also associated with the survival of patients with COAD. Their association with clinical stage, T stage, M stage, and N stage also suggests that they may contribute to the malignant development of COAD. Their roles and mechanisms in tumors are still unknown, and we look forward to future experimental studies on their roles in COAD that will show us their mechanisms in COAD and their role in tumor immune response, which we believe will be helpful and complementary to tumor immunotherapy.

## 5. Conclusion

This study identified 329 immune-related lncRNAs in colon adenocarcinoma. And 34 prognosis-related lncRNAs were obtained by univariate Cox regression. The risk score calculated by 14 immune-related lncRNAs was identified as an independent prognostic factor. Compared with clinical stages, T stages, N stages, M stages, and age, the risk score is the best in predicting poor prognosis of patients. The identification of prognostic immune-related lncRNAs and the discovery of the relationship between these lncRNAs and prognosis of COAD may provide a new direction for future research and provide a new target for a molecular targeted therapy of COAD.

## Figures and Tables

**Table 1:** The prognosis model of COAD patients was constructed with immune-related lncRNA based on multivariate Cox regression.

ID	coef	HR
AC040977.1	0.487058	1.627521
AC009237.14	0.372021	1.450663
EIF3J-AS1	0.630542	1.878628
LINC02474	0.450276	1.568745
AL513550.1	0.605145	1.831517
AC156455.1	0.356569	1.42842
AL391422.3	0.550559	1.734222
AC004080.1	0.348324	1.416691
AC008741.2	-1.07714	0.340569
LINC01503	0.614045	1.847891
AL161729.4	0.744444	2.105272
AC092687.3	0.305246	1.356959
MIR210HG	0.513543	1.671202
AC073611.1	0.523188	1.687398

## Data Availability

The data used in this study are from open public databases, and how to obtain them has been explained in the manuscript.
